# Security Analysis and Improvement of an Anonymous Authentication Scheme for Roaming Services

**DOI:** 10.1155/2014/687879

**Published:** 2014-09-11

**Authors:** Youngsook Lee, Juryon Paik

**Affiliations:** ^1^Department of Cyber Investigation Police, Howon University, 64 3-gil, Gunsan, Jeollabuk-do 573-718, Republic of Korea; ^2^Department of Computer Engineering, Sungkyunkwan University, 2066 Seoburo, Suwon, Gyeonggido 440-746, Republic of Korea

## Abstract

An anonymous authentication scheme for roaming services in global mobility networks allows a mobile user visiting a foreign network to achieve mutual authentication and session key establishment with the foreign-network operator in an anonymous manner. In this work, we revisit He et al.'s anonymous authentication scheme for roaming services and present previously unpublished security weaknesses in the scheme: (1) it fails to provide user anonymity against any third party as well as the foreign agent, (2) it cannot protect the passwords of mobile users due to its vulnerability to an offline dictionary attack, and (3) it does not achieve session-key security against a man-in-the-middle attack. We also show how the security weaknesses of He et al.'s scheme can be addressed without degrading the efficiency of the scheme.

## 1. Introduction

As wireless network and communication technologies advance, there has been a dramatic increase in the use of lightweight computing devices, such as sensors, smart phones, and tablet PCs, being used in our daily lives. To enjoy the convenience of mobility, a roaming service should be seamlessly provided with respect to availability and security, by means of using a visited foreign network. In general, three parties—a mobile user, a foreign agent, and the home agent—participate in a roaming process. A seamless roaming service requires significant security challenges to be addressed among the participants. Basically, authentication and key establishment between the mobile user and the foreign agent should be achieved via assistance of the home agent to prevent illegal usages of the network and to protect their subsequent communications. Achieving anonymity of the mobile user is also important in a roaming service to protect the privacy of the user. Anonymity has recently been identified as a major security property for many applications, including location-based services, anonymous web browsing, and e-voting. These security challenges and their cryptographic solutions, commonly called* anonymous authentication schemes*, constitute an active research area.

The first anonymous authentication scheme for roaming services was proposed by Zhu and Ma [1] in 2004. This initial proposal has been followed by a number of authentication schemes offering various levels of security and efficiency. Some schemes [2–4] have been proven secure using a computer security approach while others (e.g., [5–7]) justify their security on purely heuristic grounds without providing no formal analysis of security. However, despite all the work conducted over the last decade, it still remains a challenging task to come up with an authentication scheme that meets all the desired goals for roaming services [8]. Most of the existing schemes fail to achieve important security properties such as user anonymity [2, 6], session-key security [9], perfect forward secrecy [10], two-factor security [11], resistance against impersonation attacks [12], and resistance against offline dictionary attacks [13]. For this domain, all published schemes are far from ideal as evidenced by a continual history of schemes being proposed and years later found to be flawed.

Recently, Xie et al. [4] proposed a new authentication scheme for roaming services and claimed that their scheme not only provides efficiency and user friendliness but also is secure against various attacks. But He et al. [12] demonstrated that Xie et al.'s scheme is susceptible to impersonation attacks and therefore does not achieve mutual authentication between a mobile user and the foreign agent. In addition, He et al. proposed a new authentication scheme which improves Xie et al.'s scheme in terms of both security and efficiency. However, we found that He et al.'s improved scheme is not satisfactory enough but still suffers from major security weaknesses.

- He et al.'s scheme does not provide user anonymity not only against the foreign agent but also against any third party.
- He et al.'s scheme may not protect the passwords of mobile users against an offline dictionary attack.
- He et al.'s scheme is not secure against a man-in-the-middle attack and thus cannot guarantee the security of session keys.

Besides reporting these weaknesses in He et al.'s scheme, we also propose an improved authentication scheme which achieves, among others, user anonymity, session-key security, and resistance against offline dictionary attacks. The performance of our scheme is similar to that of He et al.'s scheme but is superior to that of Xie et al.'s scheme (see Section 4).

Throughout the paper, we make the following assumptions on the capabilities of the probabilistic polynomial-time adversary in order to properly capture security requirements of two-factor authentication schemes using smart cards in global mobility networks.

- The adversary has the complete control of all message exchanges between the three parties: a mobile user, the foreign agent, and the home agent. That is, the adversary can eavesdrop, insert, modify, intercept, and delete messages exchanged among the parties at will [14–16].
- The adversary is able to (1) extract the sensitive information on the smart card of a mobile user possibly via a power analysis attack [17, 18] or (2) learn the password of the mobile user through shoulder surfing or by employing a malicious card reader. However, it is not allowed that the adversary compromises both the information on the smart card and the password of the mobile user; it is clear that there is no way to prevent the adversary from impersonating the mobile user if both factors are compromised.

## 2. A Review of He et al.'s Scheme

He et al.'s authentication scheme [12] consists of three phases: the registration phase, the login and key agreement phase, and the password update phase. The system parameters listed in Table 1 are assumed to have been established in advance before the scheme is used in practice. Let || and ⊕ denote the string concatenation operation and the bitwise exclusive-OR (XOR) operation, respectively.

### 2.1. Registration Phase

For a mobile user *MU*, this phase is performed only once when *MU* registers itself with the home agent *HA*.

1. *MU* chooses its identity *ID*_*MU*_ and password *pw*_*MU*_ freely and sends the identity *ID*_*MU*_ to *HA* via a secure channel.
2. *HA* computes *S* *ID*_*MU*_ = *E*_*x*_(*ID*_*MU*_||*ID*_*HA*_) and *D* *ID*_*MU*_ = *H*(*ID*_*MU*_)^*x*^mod⁡*p* and issues *MU* a smart card loaded with {*S* *ID*_*MU*_, *D* *ID*_*MU*_, *ID*_*HA*_, *p*, *q*, (*E*, *D*), *H*}.
3. *MU* replaces *S* *ID*_*MU*_ and *D* *ID*_*MU*_, which are contained in the smart card, with *T* *ID*_*MU*_ = *S* *ID*_*MU*_ ⊕ *H*(0||*pw*_*MU*_) and *E* *ID*_*MU*_ = *D* *ID*_*MU*_ ⊕ *H*(1||*pw*_*MU*_), respectively.

### 2.2. Login and Key Agreement Phase

This phase is carried out whenever *MU* visits a foreign network and wants to gain access to the network. During the phase, mutual authentication and session-key establishment are conducted between *MU* and *FA* with the help of *HA*.  Algorithm 1 depicts how the phase works, and its description follows.

**Step 1 .**

*MU* inserts its smart card into the card reader and inputs its identity *ID*_*MU*_ and password *pw*_*MU*_. Next, *MU* retrieves the current timestamp *T*_1_, chooses a random number *a* ∈ *Z*_q_*, and computes

(1) A=H(IDMU)amod⁡p,KMH=(EIDMU⊕H(1||pwMU))amod⁡p=H(IDMU)axmod⁡p,kMH=H(KMH||T1),SIDMU=TIDMU⊕H(0||pwMU)=Ex(IDMU||IDHA),CMH=EkMH(SIDMU||IDMU||IDFA).

Then, *MU* sends the message *M*_1_ = 〈*ID*_*HA*_, *T*_1_, *A*, *C*_*MH*_〉 to the foreign agent *FA*.

**Step 2 .**

Upon receiving *M*_1_, *FA* checks the freshness of the timestamp *T*_1_. If it is not fresh, *FA* aborts the session. Otherwise, *FA* retrieves the current timestamp *T*_2_, computes

(2) $$\begin{array}{c} C_{FH}=E_{k_{HF}}\left({ID}_{FA}||T_{2}||M_{1}\right) \end{array}$$

and sends the message *M*_2_ = 〈*ID*_*FA*_, *T*_2_, *C*_*FH*_〉 to *HA*.

**Step 3 .**

*HA* checks if the timestamp *T*_2_ is fresh. If not, *HA* aborts the session. Otherwise, *HA* decrypts *C*_*FH*_ with key *k*_*HF*_ and verifies that the decryption yields the same *ID*_*FA*_ and *T*_2_ as contained in *M*_2_. *HA* aborts if the verification fails. Otherwise, *HA* computes *K*_*MH*_ = *A* ^*x*^mod⁡*p* and *k*_*MH*_ = *H*(*K*_*MH*_||*T*_1_), decrypts *C*_*MH*_ with key *k*_*MH*_, and checks if this decryption produces the same *ID*_*FA*_ as in *M*_2_. *HA* aborts if the check fails. Otherwise, *HA* decrypts *S* *ID*_*MU*_ with key *x* and checks if this decryption gives the same *ID*_*MU*_ as produced through the decryption of *C*_*MH*_. If only the two IDs match, *HA* retrieves the current timestamp *T*_3_, computes

(3) σ=H(M1||T3||KMH||IDMU||IDFA||IDHA),CHF=EkHF(IDMU||IDFA||T3||σ),

and sends the message *M*_3_ = 〈*ID*_*FA*_, *T*_3_, *C*_*HF*_〉 to *FA*.

**Step 4 .**

*FA* decrypts *C*_*HF*_ with key *k*_*HF*_ and checks the freshness of the timestamp *T*_3_. If only *T*_3_ is fresh, *FA* chooses a random number *b* ∈ *Z*_q_* and computes

(4) B=H(IDMU)bmod⁡p,KFM=Abmod⁡p=H(IDMU)abmod⁡p,kFM=H(KFM),CFM=EkFM(IDMU||IDFA||T3||σ||B).

(Note, here, that the timestamp *T*_3_ (received from *HA*) is used in generating the ciphertext *C*_*FM*_ since *MU* will need it to check the validity of *σ*.) Then, *FA* sends the message *M*_4_ = 〈*ID*_*FA*_, *T*_3_, *B*, *C*_*FM*_〉 to *MU* and computes the session key *sk* = *H*(*K*_*FM*_ + 1).

**Step 5 .**

*MU* first checks the freshness of the timestamp *T*_3_ and aborts the session if not fresh. Otherwise, *MU* computes *K*_*FM*_ = *B* ^*a*^mod⁡*p* and *k*_*FM*_ = *H*(*K*_*FM*_), decrypts *C*_*FM*_ with key *k*_*FM*_, and verifies that the decryption correctly returns *ID*_*MU*_, *ID*_*FA*_, and *T*_3_. If the verification succeeds, *MU* checks if *σ* is equal to *H*(*M*_1_||*T*_3_||*K*_*MH*_||*ID*_*MU*_||*ID*_*FA*_||*ID*_*HA*_) and if equal computes the session key *sk* = *H*(*K*_*FM*_ + 1).

### 2.3. Password Update Phase

One of the general guidelines to get better password security is to ensure that passwords are changed at regular intervals. He et al.'s scheme allows mobile users to freely update their passwords.

1. *MU* inserts his smart card into a card reader and enters both the current password *pw*_*MU*_ and the new password *pw*_*MU*_′.
2. The smart card computes *T* *ID*_*MU*_′ = *T* *ID*_*MU*_ ⊕ *H*(0||*pw*_*MU*_) ⊕ *H*(0||*pw*_*MU*_′) and *E* *ID*_*MU*_′ = *E* *ID*_*MU*_ ⊕ *H*(1||*pw*_*MU*_) ⊕ *H*(1||*pw*_*MU*_′) and replaces *T* *ID*_*MU*_ and *E* *ID*_*MU*_ with *T* *ID*_*MU*_′ and *E* *ID*_*MU*_′, respectively.

## 3. Weaknesses in He et al.'s Scheme

In this section, we point out four weaknesses in He et al.'s scheme, starting with the most obvious one. 

*Weakness  1*. He et al.'s scheme does not provide user anonymity against the foreign agent *FA*.

This weakness is straightforward to see as the identity of *MU*, *ID*_*MU*_, is given to *FA* via the ciphertext *C*_*HF*_ (see Step 4 of the login and key agreement phase of the scheme). 

*Weakness  2*. He et al.'s scheme may not protect the password of *MU*, *pw*_*MU*_, against an offline dictionary attack.

Weakness 2 is due to the fact that *E* *ID*_*MU*_ is computed using the bitwise XOR operation when the multiplicative subgroup of *Z*_p_* is not closed under the XOR operation. This design flaw allows an adversary to find out the password *pw*_*MU*_ by mounting an offline dictionary attack if the subgroup is much smaller than *Z*_p_*. We observe, for He et al.'s scheme, that (1)*p* and *q* are defined as two primes such that *p* = *rq* + 1 for some *r* ∈ *N* and (2) the random exponents *a* and *b* are chosen from *Z*_q_*. Based on these observations, it is reasonable to speculate that He et al.'s scheme was designed to work in a multiplicative subgroup of *Z*_p_* that has a prime order *q*, though not explicitly mentioned by the authors. For simplicity, let us denote the prime-order subgroup by *G*. Since *K*_*MH*_ and *D* *ID*_*MU*_ are computed as *K*_*MH*_ = (*D* *ID*_*MU*_)^*a*^mod⁡*p* and *D* *ID*_*MU*_ = *H*(*ID*_*MU*_)^*x*^mod⁡*p*, it ought to be the case that *D* *ID*_*MU*_ ∈ *G*, which in turn implies that *H* is a hash function mapping arbitrary strings into elements of *G*. Now, assume that an adversary *A* has gained temporary access to the smart card of *MU* and then obtained the value of *E* *ID*_*MU*_ stored there (possibly by employing a power analysis attack [17]). Then, note that *E* *ID*_*MU*_ can be used as a password verifier in an offline dictionary attack because *E* *ID*_*MU*_ is computed as *E* *ID*_*MU*_ = *D* *ID*_*MU*_ ⊕ *H*(1||*pw*_*MU*_) when *G* is not closed under the bitwise XOR operation. Let *PW* be the set of all possible passwords. The adversary *A* can mount an offline dictionary attack as follows.

**Step 1 .**

*A* makes a guess *pw*_*MU*_′ ∈ *PW* on the password *pw*_*MU*_ and computes

(5) $$\begin{array}{c} {DID}_{MU}^{\prime }=EID_{MU}⊕H\left(1||{pw}_{MU}^{\prime }\right). \end{array}$$

**Step 2 .**

*A* then checks whether *D* *ID*_*MU*_′ is an element of *G* or not. If *D* *ID*_*MU*_′ ∉ *G*, *A* deletes *pw*_*MU*_′ from the dictionary *PW* (i.e., *PW* = *PW*∖{*pw*_*MU*_′}). Note that *D* *ID*_*MU*_′ ∉ *G* implies *pw*_*MU*_′ ≠ *pw*_*MU*_.

**Step 3 .**

*A* repeats Steps 1 and 2 until the correct password is found (i.e., until |*PW* | = 1).

If *p* is a safe prime (i.e., *p* = 2*q* + 1), then this attack would fail, cutting only the size of *PW* about in half. However, if *p* is much greater than *q* (e.g., log⁡_2_ *p*⋍512 and log⁡_2_ *q*⋍256), the dictionary attack will succeed in determining the correct password with an overwhelming probability. Similar dictionary attacks have been also mounted against key exchange protocols; see, for example, [19]. Weakness 2 can be easily addressed by replacing the bitwise XOR operation with the multiplication operation.

Next, we identify two other major weaknesses in He et al.'s scheme.

*Weakness  3*. He et al.'s scheme may not guarantee user anonymity even against a third party who is not a legitimate protocol participant. 

*Weakness  4*. He et al.'s scheme could wrongly lead *MU* and *FA* to establish a session key with a malicious party who is not even registered with *HA*. 

**Figure 1:**
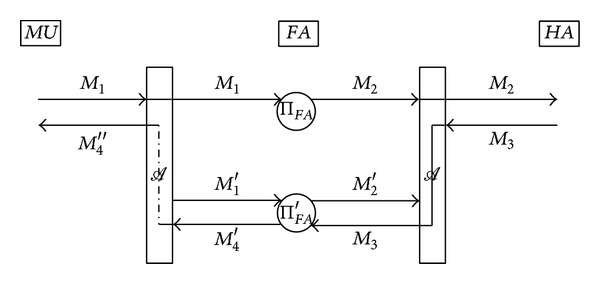
A man-in-the-middle attack on He et al.'s scheme.

We demonstrate Weaknesses  3 and 4 by mounting a type of man-in-the-middle attack against the scheme. The attack scenario is outlined in Figure 1 and is detailed as follows.

**Step 1 .**

As a preliminary step, the adversary *A* chooses a random number *a*′ ∈ *Z*_q_* and computes *A*′ = *H*(*ID*)^*a*′^mod⁡*p*, where *ID* denotes an arbitrary identity.

**Step 2 .**

When *MU* sends the first message *M*_1_ = 〈*ID*_*HA*_, *T*_1_, *A*, *C*_*MH*_〉 to *FA*, *A* eavesdrops on this message to obtain *A* and *C*_*MH*_. Immediately after the eavesdropping, *A* retrieves the current timestamp *T*_1_′ and sends a fake message *M*_1_′ = 〈*ID*_*HA*_, *T*_1_′, *A*′, *C*_*MH*_〉 to *FA* as if it is another roaming request from a mobile user.

**Step 3 .**

Since both *T*_1_ and *T*_1_′ are fresh, *FA* will compute *C*_*FH*_ = *E*_*k*_*HF*__(*ID*_*FA*_||*T*_2_||*M*_1_) and *C*_*FH*_′ = *E*_*k*_*HF*__(*ID*_*FA*_||*T*_2_′||*M*_1_′) and send two messages *M*_2_ = 〈*ID*_*FA*_, *T*_2_, *C*_*FH*_〉 and *M*_2_′ = 〈*ID*_*FA*_, *T*_2_′, *C*_*FH*_′〉 to *HA*. Let Π_*FA*_ and Π_*FA*_′ be the instances of *FA* who sends the messages *M*_2_ and *M*_2_′, respectively.

**Step 4 .**

*A* intercepts the message *M*_2_′ while letting *M*_2_ reach its destination, *HA*. Since *M*_2_ is a valid message, *HA* will compute

(6) σ=H(M1||T3||KMH||IDMU||IDFA||IDHA),CHF=EkHF(IDMU||IDFA||T3||σ),

and send the message *M*_3_ = 〈*ID*_*FA*_, *T*_3_, *C*_*HF*_〉 to *FA*.

**Step 5 .**

*A* redirects the message *M*_3_ so that it is delivered to Π_*FA*_′ instead of Π_*FA*_. As a result, Π_*FA*_ will not receive any response message and thus will abort after a certain amount of time.

**Step 6 .**

After decrypting *C*_*HF*_ and since *T*_3_ is fresh, Π_*FA*_′ will proceed as per the protocol specification. That is, Π_*FA*_′ will choose a random number *b*′ ∈ *Z*_q_*, compute

(7) B′=H(IDMU)b′mod⁡p,KFM′=A′b′mod⁡p=H(ID)a′b′mod⁡p,kFM′=H(KFM′),CFM′=EkFM′(IDMU||IDFA||T3||σ||B′),

send the message *M*_4_′ = 〈*ID*_*FA*_, *T*_3_, *B*′, *C*_*FM*_′〉 to *MU*, and then compute its session key as

(8) $$\begin{array}{c} sk_{FA}=H\left(K_{FM}^{\prime }+1\right). \end{array}$$

**Step 7 .**

*A* intercepts the message *M*_4_′, computes *K*_*FM*_′ = *B*′^*a*′^mod⁡*p* and *k*_*FM*_′ = *H*(*K*_*FM*_′), and decrypts *C*_*FM*_′ with key *k*_*FM*_′ to obtain *ID*_*MU*_, *ID*_*FA*_, and *σ*. Then, *A* chooses a random number *b*′′ ∈ *Z*_q_*, computes

(9) B′′=H(IDMU)b′′mod⁡p,KFM′′=Ab′′mod⁡p=H(IDMU)ab′′mod⁡p,kFM′′=H(KFM′′),CFM′′=EkFM′′(IDMU||IDFA||T3||σ||B′′),

and sends the message *M*_4_′′ = 〈*ID*_*FA*_, *T*_3_, *B*′′, *C*_*FM*_′′〉 to *MU* as if it is from *FA*.

**Step 8 .**

Upon receiving *M*_4_′′, *MU* will proceed to compute its session key

(10) $$\begin{array}{c} sk_{MU}=H\left(K_{FM}^{\prime \prime }+1\right), \end{array}$$

where *K*_*FM*_′′ is computed as *K*_*FM*_′′ = *B* ^′′*a*^mod⁡*p*, because (1) *T*_3_ is fresh, (2) decryption of *C*_*FM*_′′ with key *k*_*FM*_′′ correctly yields *ID*_*MU*_, *ID*_*FA*_, and *T*_3_, and (3) *σ* is equal to *H*(*M*_1_||*T*_3_||*K*_*MH*_||*ID*_*MU*_||*ID*_*FA*_||*ID*_*HA*_).

**Step 9 .**

*A* computes the two session keys, *sk*_*FA*_ and *sk*_*MU*_, in the straightforward way.

Through the attack, user anonymity is completely compromised as the identity of *MU*, *ID*_*MU*_, is disclosed to the adversary *A* in Step 7. From the viewpoint of session-key secrecy, the effect of our attack is the same as that of a man-in-the-middle attack. At the end of the attack, *MU* and *FA* believe that they have established a secure session with each other sharing a secret key, while in fact they have shared their keys with the adversary *A*. As a result, *A* can not only access and relay any confidential messages between *MU* and *FA* but also send arbitrary messages for its own benefit impersonating one of them to the other. Man-in-the-middle attacks similar to the attack above have been also presented against various key exchange protocols; see, for example, [20, 21].

## 4. Our Improved Scheme

We now show how to address all the weaknesses identified in He et al.'s scheme without degrading the efficiency of the scheme. Let *G* be a cyclic group of prime order *q*. A standard way of generating *G* is to choose two large primes *p*, *q* such that *p* = *rq* + 1 for some small *r* ∈ *N* (e.g., *r* = 2) and let *G* be the subgroup of order *q* in *Z*_p_*. Hereafter, we will omit “mod *p*” from expressions for notational simplicity. Assume that the master secret key of *HA*, *x*, is an element of *Z*_q_* (i.e., *x* ∈ *Z*_q_*) and the secret key shared between *FA* and *HA*, *k*_*HF*_, has length of *l* bits. Then we define four cryptographic hash functions:

- *F* : {0,1}* → {0,1}^*l*^,
- *G* : {0,1}* → *G*,
- *H* : {0,1}* → {0,1}^*κ*^, where *κ* represents the bit-length of session keys,
- *I* : {0,1}* → {0,1}^*ɛ*^, where *ɛ* represents the bit-length of *S* *ID*_*MU*_ (for the definition of *S* *ID*_*MU*_, see the description of He et al.'s scheme given in Section 2).

We begin by presenting how to address Weaknesses  3 and 4 (described in the previous section). The vulnerability of He et al.'s scheme to the man-in-the-middle attack is because there is no way for an instance of *FA* to check whether the received ciphertext *C*_*HF*_ was sent in response to its own request or another instance's request. This design flaw allows the adversary to exploit *HA*'s response sent for one session as the response for another session. To prevent the attack, we suggest to modify the computation of the ciphertext *C*_*HF*_ from *C*_*HF*_ = *E*_*k*_*HF*__(*ID*_*MU*_||*ID*_*FA*_||*T*_3_||*σ*) to

(11) $$\begin{array}{c} C_{HF}=E_{k_{HF}}\left(ID_{MU}||ID_{FA}||T_{2}||T_{3}||\sigma \right). \end{array}$$

The timestamp *T*_2_ is now included as part of the plaintext to be encrypted to *C*_*HF*_. The inclusion of *T*_2_ tightly links *FA*'s request and *HA*'s response and thus effectively prevents the man-in-the-middle attack.

However, with the above modification alone, He et al.'s scheme cannot fully achieve user anonymity in the sense that the identity of *MU* is still disclosed to *FA*. Therefore, we suggest to further modify the computation of *C*_*HF*_ as follows:

(12) $$\begin{array}{c} C_{HF}=E_{k_{HF}}\left(G\left(ID_{MU}\right)||ID_{FA}||T_{2}||T_{3}||\sigma \right). \end{array}$$

The ciphertext *C*_*HF*_ is now generated using *G*(*ID*_*MU*_) instead of *ID*_*MU*_. This modification certainly prevents *FA* from immediately learning *ID*_*MU*_ via decryption of *C*_*HF*_.

We next present a possible way of eliminating the vulnerability of He et al.'s scheme to offline dictionary attacks. Recall that this vulnerability is due to the fact that *E* *ID*_*MU*_ is computed using the bitwise XOR operation when the multiplicative subgroup of *Z*_p_* is not closed under the XOR operation. Given the flaw in the design, the solution is clear; use the multiplication operation instead of the XOR operation when computing *E* *ID*_*MU*_. Hence, we change the computation of *E* *ID*_*MU*_ from *E* *ID*_*MU*_ = *D* *ID*_*MU*_ ⊕ *H*(1||*pw*_*MU*_) to

(13) $$\begin{array}{c} EID_{MU}=DID_{MU}\cdot G{\left(1||pw_{MU}\right)}^{-1}. \end{array}$$

Accordingly, the computation of *K*_*MH*_ should be also changed to

(14) KMH=(EIDMU·G(1||pwMU))a=G(IDMU)ax.

Finally, we suggest the following additional changes to resolve some notational ambiguities and to correct the misuse of the hash function *H*:

(15) SIDMU=EF(x)(IDMU||IDHA),  DIDMU=G(IDMU)x,TIDMU=SIDMU⊕I(0||pwMU)A=G(IDMU)a,kMH=F(KMH||T1),SIDMU=TIDMU⊕I(0||pwMU),B=G(IDMU)b,  kFM=F(KFM).

As a result of the above modifications, the password update phase is modified as follows.

1. *MU* inserts his smart card into a card reader and enters the identity *ID*_*MU*_, the current password *pw*_*MU*_, and the new password *pw*_*MU*_′.
2. The smart card computes *T* *ID*_*MU*_′ = *T* *ID*_*MU*_ ⊕ *I*(0||*pw*_*MU*_) ⊕ *I*(0||*pw*_*MU*_′) and *E* *ID*_*MU*_′ = *E* *ID*_*MU*_ · *G*(1||*pw*_*MU*_) · *G*(1||*pw*_*MU*_′)^−1^ and replaces *T* *ID*_*MU*_ and *E* *ID*_*MU*_ with *T* *ID*_*MU*_′ and *E* *ID*_*MU*_′, respectively.

Combining the above modifications together yields an improved authentication scheme described in Algorithm 2. Our scheme improves He et al.'s scheme in various aspects: (1) it enjoys the anonymity of the mobile user *MU* against any parties other than the home agent *HA*, including the foreign agent *FA*; (2) it withstands offline dictionary attacks even when the information in the smart card is disclosed; (3) it protects the security of session keys against man-in-the-middle attacks. Clearly, the performance of our scheme is similar to that of He et al.'s scheme. Hence, we can say that our improvement enhances the security of He et al.'s scheme while maintaining the efficiency of the scheme.

## 5. Concluding Remarks

This work demonstrated that He et al.'s authentication scheme for roaming services fails to achieve major security properties—user anonymity, password security, and session-key security—in the presence of a malicious adversary. We have shown that failure to achieving user anonymity and session-key security is due to the vulnerability to a man-in-the-middle attack while failure to achieving password security is due to the vulnerability to an offline dictionary attack. Note that the latter vulnerability implies that He et al.'s scheme does not achieve two-factor security. We hope that similar security flaws as identified in this work can be prevented in the future design of anonymous authentication schemes.

This work also showed how the security of He et al.'s authentication scheme can be improved without efficiency degradation. Our improved scheme not only protects user anonymity against any third parties other than the home agent but also is secure against offline dictionary attacks as well as man-in-the-middle attacks. We leave it as a future work to design an anonymous authentication scheme for roaming services that achieves provable security in a well-defined communication model while providing the same (or even better) level of efficiency as the schemes studied in this paper.

## Figures and Tables

**Algorithm 1:**
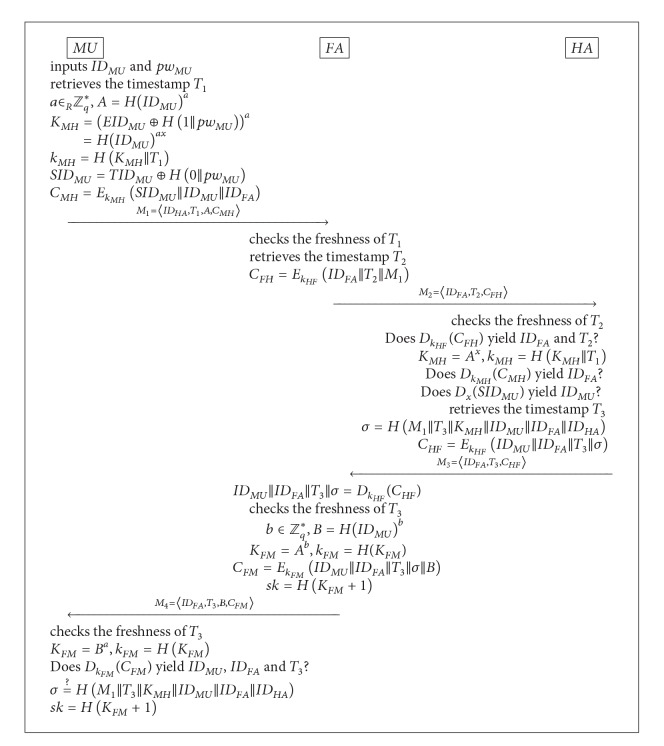
Login and key agreement phase of He et al.'s scheme [12].

**Algorithm 2:**
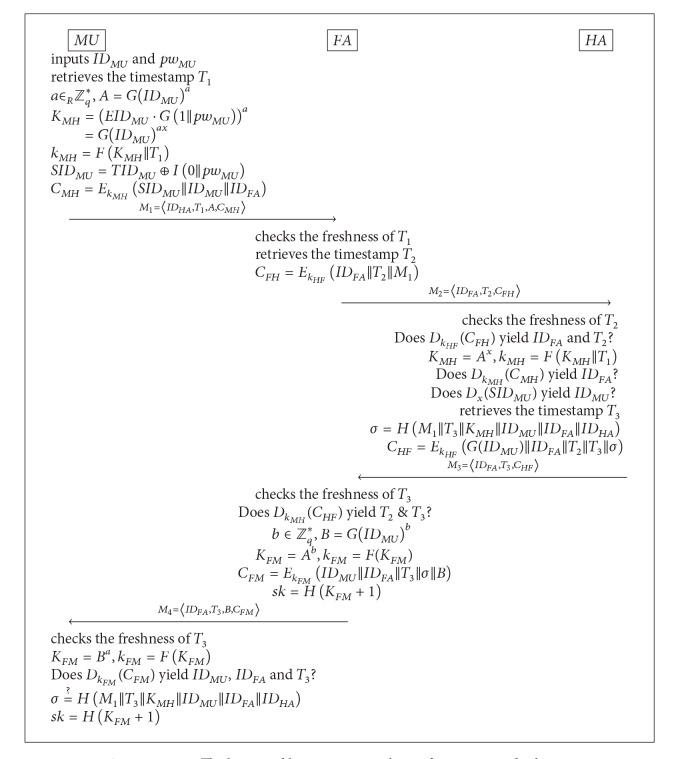
The login and key agreement phase of our improved scheme.

**Table 1:** System parameters.

*ID*_*HA*_, *ID*_*FA*_	The identities of *HA* and *FA*, respectively
*p*, *q*	Two large primes such that *p* = *rq* + 1 for some *r* ∈ *N*
*x*	The master secret key of *HA*
*k*_*HF*_	A (cryptographically strong) key shared between *HA* and *FA*
(*E*, *D*)	A pair of symmetric encryption and decryption algorithms
*H*(·)	A cryptographic hash function
